# Supplementary material for: A Controlled, Retrospective, Single-Center Study to Evaluate the Role of a Probiotic Mixture Administered during Pregnancy in Reducing Streptococcus Agalactiae Swab Positivity and the Frequency of Premature Rupture of Amniochorionic Membranes
Source: Microorganisms. 2024 Sep 30;12(10):1979. doi: 10.3390/microorganisms12101979 (PMC11509217; doi:10.3390/microorganisms12101979)
Supplement: Supplementary file 1 [file microorganisms-12-01979-s001.zip › microorganisms-3240285-supplementary.pdf]

Supplementary File S1. Number of patients globally involved in our retrospective analysis.

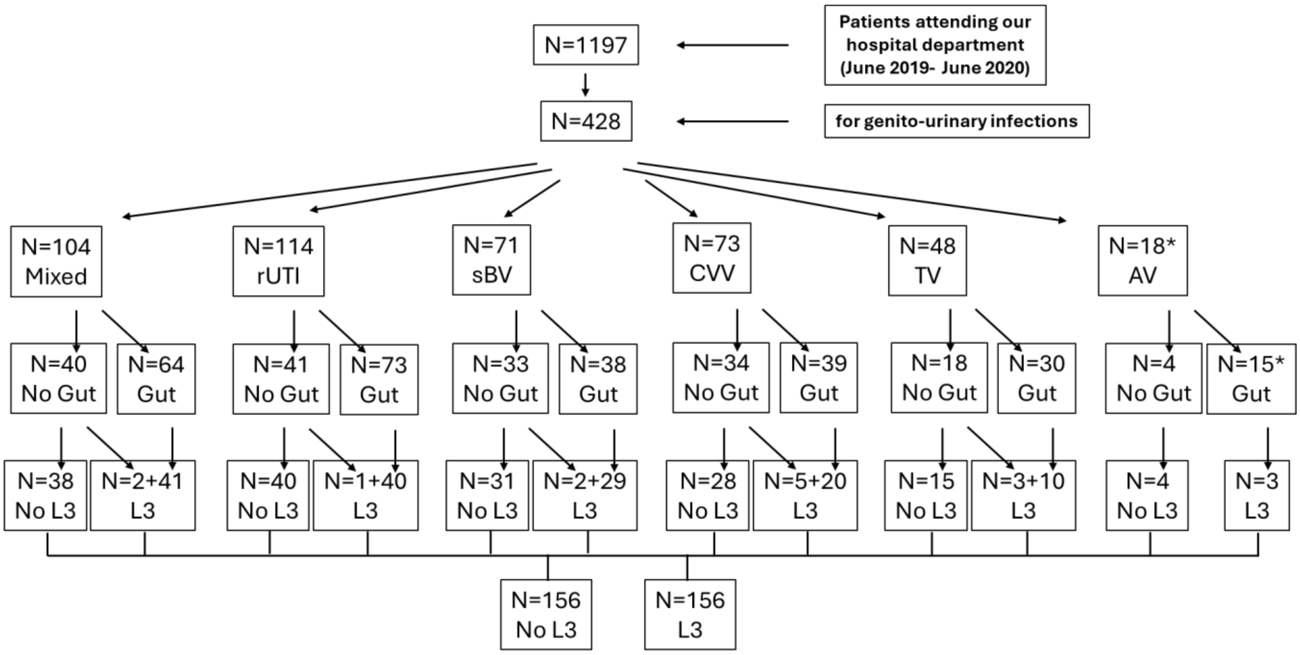

Mixed: mixed infection (bladder + vaginal); rUTI: recurrent Urinary Tract Infection); sBV; symptomatic Bacterial Vaginosis; CVV: Candida Vulvo-Vaginitis; TV: Trichomonas vaginalis; AV: Aerobic Vaginitis (*:11 menopausal women); No Gut: patients not declaring gut discomfort; Gut: patients declaring gut discomfort; No L3: not treated with the L3-based probiotic; L3: treated with L3-based probiotic.

Supplementary File S2. Labor induction method.

In the case of PROM and in the absence of spontaneous labor, the method adopted for labor induction coincided with the administration of dinoprostone. In fact, dinoprostone allows to induce the maturation of the uterine cervix with changes in consistency, dilation and cervical flattening. Indirectly, dinoprostone facilitates the induction of uterine contractile activity and therefore stimulates the myometrial response to the effects of endogenous or exogenous oxytocin. Dinoprostone was used in the form of a vaginal device (Propess®) with controlled release of 10 mg (0.3 mg/h). The controlled release device has the advantage of being easily removed if labor begins or if hypertonicity or tachysystole occur (on average in 5 -15% of women) or other adverse events. In these cases, the effect of the drug disappears within 15 minutes of removal. A further advantage of the vaginal device is that the modulated and constant release of the drug leads to a greater and progressive induction of cervical maturation and labor. For these reasons, in the case of PROM and in the absence of spontaneous labor, it was decided to use the administration of dinoprostone in the formulation of a controlled-release vaginal device as an induction method. During induction with prostaglandins, cardiotocographic monitoring was also performed for 20-40 minutes until fetal reactivity was achieved before applying the drug and subsequently monitoring for approximately one hour. The vaginal device was removed after a maximum of 24 hours from administration.

Supplementary file S3. Sample size calculation.

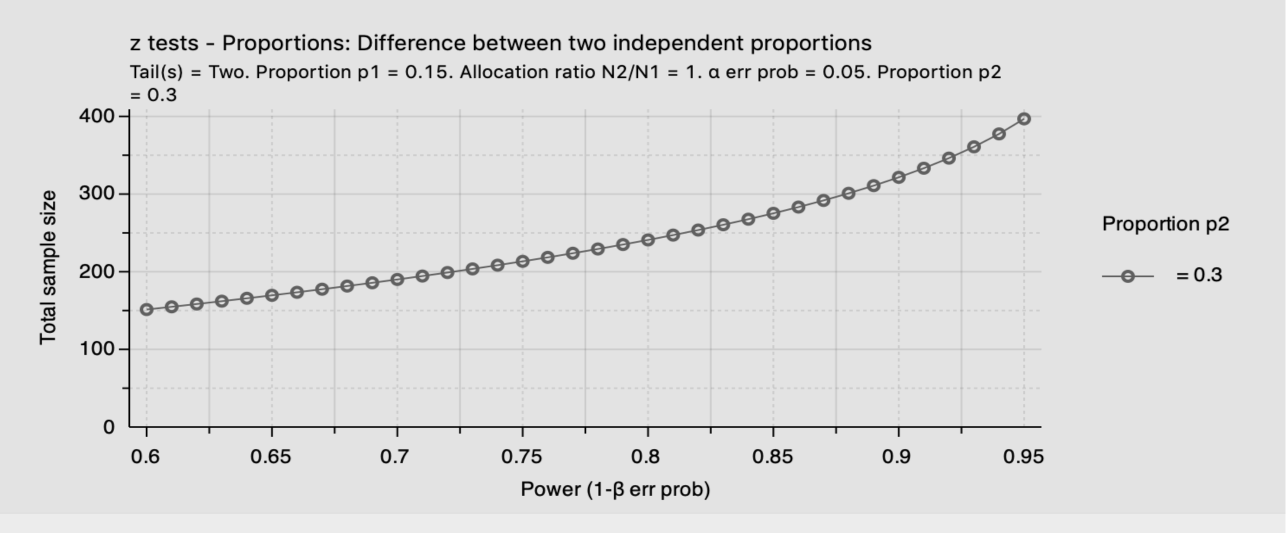

Group sample sizes of 121 in group one and 121 in group two achieve 80% power to detect a difference between the group proportions of 0.1500. The proportion in group one (the treatment group) is assumed to be 0.1500 under the null hypothesis and 0.3000 under the alternative hypothesis. The proportion in group two (the control group) is 0.3000. The test statistic used is the two-sided Z test with pooled variance. The significance level of the test was targeted at 0.0500 (Reference: Fleiss, J. L., Levin, B., and Paik, M.C. 2003. Statistical Methods for Rates and Proportions. Third Edition. John Wiley & Sons. New York).

Supplementary File S4. Reasons for exclusion from the retrospective evaluation. Number of excluded patients are reported into brackets.

Main reason	Group A (N=156)<br>Probiotic-treated	Group B (N=156)<br>Control group
**Treatment**	Treatment with other probiotic or prebiotic products (22);<br>adherence to probiotic therapy inferior to 75% (3);	Treatment with probiotic products (27)
**Failure to fully participate in the established protocol**	Willingness<br>to discontinue study (1);<br>failure to be available at the end of the study (5).	Failure to be available at the end of the study (5).

The differences observed in the values between the two groups are not significant.

Supplementary file S5. *Taxa* involved in urine culture positivity observed in the two groups. Numbers represent patients.

	Group A (N=34)<br>Probiotic-treated	Group B (N=37)<br>Control group
E. coli	21	23
E. faecalis	7	5
E. faecium	4	5
***Klebsiella* spp.**	2	3
**Others or mixed**	0	1

The differences observed in the values between the two groups are not significant.

Supplementary File S6. Percentage of women delivering by cesarean section in the two study groups.

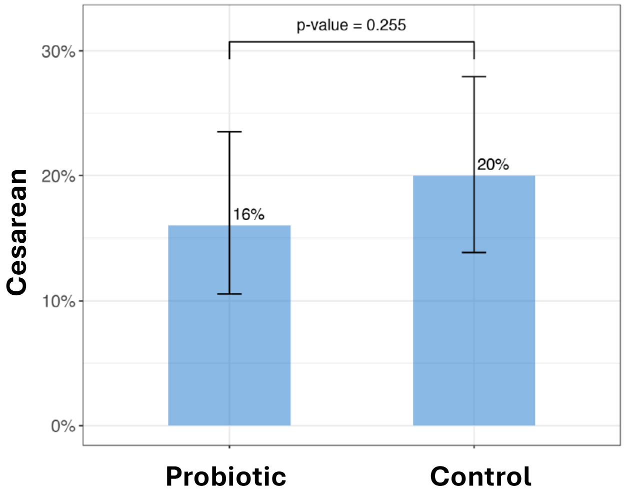

In the probiotic group, the value of 16% corresponds to 20 women. In the control group, the value of 20% corresponds to 25 women. Of course, the remaining number of women of each group, 105 for the probiotic group and 100 for the control one, delivered naturally. The differences observed in the values between the two groups are not significant.

Supplementary file S7. Unexpected, opposite or different to the patient history, gut and extra-gut side effects recorded during the 12 weeks of treatment and in the following 12 weeks. Number of patients are shown.

	Group A (N=125) Probiotic-treated	Group B (N=125) Control group
**Constipation (C)**	12	15
**Severity of C***	6 (I); 6 (II)	5 (I); 10 (II)
**Diarrhea (D)**	6	9
**Severity of D***	4 (I); 2 (II)	6 (I); 3 (II)
**Bloating (B)**	18	21
**Severity of B***	10 (I); 8 (II)	11 (I); 10 (II)
**Gastric discomfort**	7	11
**Migraine/headache**	2/6	3/5
**Mild insomnia**	11	14
**Skin rash**	2	5

*The numbers from I to IV indicate the severity of the disorder (where I is mild and IV is severe). The differences observed in the values between the two groups are not significant.

Supplementary File S8. Intrapartum prophylaxis protocol.

In patients not allergic to penicillin, the intrapartum prophylaxis protocol involves the administration of ampicillin 2 g iv, followed by 1 g iv every 4 hours until delivery. In patients at high risk of anaphylaxis (previous allergic reaction to ampicillin with skin rash or widespread urticaria, angioedema, respiratory distress), we relayed on the antibiogram outcome and, if sensitivity was observed, administration of clindamycin at 900 mg iv every 8 hours until delivery was adopted. In the absence of this data or in case of resistance to macrolides, the administration of vancomycin at 1 g iv every 12 hours until delivery was adopted.
